# Dihydroartemisinin-piperaquine versus chloroquine to treat vivax malaria in Afghanistan: an open randomized, non-inferiority, trial

**DOI:** 10.1186/1475-2875-9-105

**Published:** 2010-04-21

**Authors:** Ghulam Rahim Awab, Sasithon Pukrittayakamee, Mallika Imwong, Arjen M Dondorp, Charles J Woodrow, Sue Jean Lee, Nicholas PJ Day, Pratap Singhasivanon, Nicholas J White, Faizullah Kaker

**Affiliations:** 1 Faculty of Tropical Medicine, Mahidol University, Bangkok, Thailand; 2 Ministry of Public Health, Islamic Republic of Afghanistan, Kabul, Afghanistan; 3 Mahidol-Oxford Tropical Medicine Research Unit (MORU), Mahidol University, Bangkok, Thailand; 4 Centre for Tropical Medicine, Churchill Hospital, Oxford, UK; 5 Division of Clinical Sciences, St. George's, University of London, UK

## Abstract

**Background:**

Afghanistan's national guidelines recommend chloroquine for the treatment of *Plasmodium vivax *infection, the parasite responsible for the majority of its malaria burden. Chloroquine resistance in *P. vivax *is emerging in Asia. Therapeutic responses across Afghanistan have not been evaluated in detail.

**Methods:**

Between July 2007 and February 2009, an open-label, randomized controlled trial of chloroquine and dihydroartemisinin-piperaquine in patients aged three months and over with slide-confirmed *P. vivax *mono-infections was conducted. Consistent with current national guidelines, primaquine was not administered. Subjects were followed up daily during the acute phase of illness (days 0-3) and weekly until day 56. The primary endpoint was the overall cumulative parasitological failure rate at day 56 after the start of treatment, with the hypothesis being that dihydroartemisinin-piperaquine was non-inferior compared to chloroquine (Δ = 5% difference in proportion of failures).

**Results:**

Of 2,182 individuals with positive blood films for *P. vivax*, 536 were enrolled in the trial. The day 28 cure rate was 100% in both treatment groups. Parasite clearance was more rapid with dihydroartemisinin-piperaquine than chloroquine. At day 56, there were more recurrent infections in the chloroquine arm (8.9%, 95% CI 6.0-13.1%) than the dihydroartemisinin-piperaquine arm (2.8%, 95% CI 1.4-5.8%), a difference in cumulative recurrence rate of 6.1% (2-sided 90%CI +2.6 to +9.7%). The log-rank test comparing the survival curves confirmed the superiority of dihydroartemisinin-piperaquine over chloroquine (p = 0.003). Multivariate analysis showed that a lower initial haemoglobin concentration was also independently associated with recurrence. Both regimens were well tolerated and no serious adverse events were reported.

**Conclusions:**

Chloroquine remains an efficacious treatment for the treatment of *vivax *malaria in Afghanistan. In a setting where radical therapy cannot be administered, dihydroartemisinin-piperaquine provides additional benefit in terms of post-treatment prophylaxis, reducing the incidence of recurrence from 4-8 weeks after treatment.

**Trial Registration:**

The trial was registered at ClinicalTrials.gov under identifier NCT00682578.

## Background

*Plasmodium vivax *is associated with less mortality than *Plasmodium falciparum*, but still exerts a considerable burden of disease globally [1] and is a major cause of morbidity in Asia [2]. In endemic areas it is a particular problem of children and pregnant women in whom it is associated with poor outcomes, notably anaemia and low birth weight [3]. Control measures are confounded by the persistence of dormant hypnozoite stages in the liver, which cause relapses of the infection if radical therapy is not administered. Chloroquine remains the treatment of choice for treatment for the erythrocytic stage of *P. vivax *infection, but chloroquine resistance is spreading [4]. High level resistance is still confined mainly to Oceania [5] and Indonesia [6], but lower level resistance has been reported in other areas across Asia and South America [4].

Chloroquine was used for more than 50 years as the first-line drug for all forms of malaria in Afghanistan. Chloroquine continues to be first-line drug treatment as a mono-therapy for slide-confirmed *P. vivax *malaria and is also used in combination with sulphadoxine-pyrimethamine for presumptive treatment of clinical malaria episodes when accurate laboratory diagnosis is unavailable. Radical treatment with primaquine is not currently recommended because of the risk of oxidant haemolysis in patients with glucose-6-phosphate dehydrogenase (G6PD) deficiency, as testing for G6PD deficiency is not available. Limited access to prompt diagnosis and treatment, self-medication with low quality medicines [7] and non-adherence to the national treatment policy are all potential contributors to drug resistance in Afghanistan. To date, anti-malarial therapeutic monitoring in Afghanistan has largely been conducted at the Malaria Reference Centre, Jalalabad (Nangarhar province) in the eastern region of the country near the border with Pakistan. In this area, chloroquine resistance is established in *P. falciparum *[8] but appears not to be a major problem for *P. vivax *[9,10]. Relatively little data exists for the rest of the country; sulphadoxine-pyrimethamine has been shown to have good efficacy against *P. falciparum *in Kunduz province [11] and molecular studies indicate that *P. vivax *resistance to sulphadoxine-pyrimethamine is present at a relatively early stage in Nangarhar and Herat provinces[12].

Since 2004, artemisinin combination therapy (ACT) in the form of artesunate/sulphadoxine-pyrimethamine has been the recommended first-line treatment for *P. falciparum *infections in Afghanistan. As the correct malaria parasite species may not be identified in patients presenting with fever, *P. vivax *infections in Afghanistan will inevitably be treated with ACT [9]. For operational reasons, there may an argument for treating both vivax and falciparum malaria with the same drug regimen. The fixed-dose combination of dihydroartemisinin co-formulated with the bisquinoline piperaquine represents a promising alternative to chloroquine. Piperaquine is related to 4-aminoquinolines but it retains activity against chloroquine-resistant *P. falciparum *[13] and several studies from Indonesia and Papua New Guinea have indicated that dihydroartemisinin-piperaquine is also safe and effective for the treatment of chloroquine-resistant *vivax *malaria, with more long-lasting post-treatment prophylactic effects than other forms of ACT [5,14,15]. Dihydroartemisinin-piperaquine is relatively expensive (1-2 US$ per treatment course) but has a simple dosing schedule (daily doses for three days).

For the purpose of evidence-based decision making, and to obtain better information on the effectiveness of available anti-malarial drugs against vivax malaria in Afghanistan, the relative therapeutic efficacy of chloroquine and dihydroartemisinin-piperaquine in the treatment of vivax malaria was assessed at three sites in Afghanistan.

## Methods

### Study area and participants

The study was conducted from July 2007 to February 2009 at three provincial malaria control centres, one in the east and two in the north of the country. The epidemiology of malaria in Afghanistan is determined by the physical geography of the country, malaria being confined to lower lying areas with sufficient rainfall for mosquito survival consisting of the northern plains (bordering Turkmenistan, Uzbekistan and Tajikistan) [16], the Jalalabad basin to the east (bordering Pakistan) and river valleys that fringe the central mountains to the west and south [17]. The central area of Afghanistan, occupied by the western end of the Hindu Kush mountain range, is relatively free of malaria [18,19]. In Afghanistan, *P. vivax *remains endemic and accounts for more than 90% of all malaria cases [20]. Cases begin to present in the spring months (May onwards) consistent with periodic relapse from long latency hypnozoites, reach a peak in July and August, and few cases are seen after November.

Jalalabad, capital of Nangarhar province, is a referral centre for the whole eastern region and lies adjacent to the Pakistani border where population movement in both directions takes place. The population is multi-ethnic with Pashtoon being the dominant group. The province as a whole (altitudes below 2,000 meters) is generally semiarid but where possible rice is cultivated. Taloqan is the capital of the north-east province of Takhar with a population that is mainly Tajik with significant proportions of Uzbek and Pashtoon. Rice is cultivated on the plain and malaria is a well-known health problem [21]. Maimana is the capital city of the north-west province of Faryab bordering Turkmenistan, with which there is no open border. The population is mainly Uzbek and Turkmen and the region is semi-arid.

### Enrolment

This was a prospective, open label, randomized controlled trial conducted in patients with microscopically confirmed symptomatic vivax malaria who were aged three months and older. All febrile patients over three months of age presenting to the sites were screened and enrolled according to preset criteria. A standard data sheet was used to record demographic information, details of symptoms and their duration, and previous anti-malarial medications. Clinical examination findings and vital signs were documented, including axillary temperature measured with a digital thermometer. Venous blood samples were obtained for haemoglobin measurement (Hemocue) and white cell count using the counting chamber method. Giemsa-staining of thick and thin blood films was performed to confirm parasite species and parasitaemia by a standard approximation method (40 × number of parasites per 200 white blood cells on the thick film). 10% of slides were cross-checked in the MORU (Bangkok) parasitology laboratory. Approximately 20 μl capillary blood was collected on filter papers for subsequent confirmation of species by PCR and future molecular studies. Slide examination results were considered to be negative after examination of 30 high-power fields of the thick film.

The inclusion criteria were slide-confirmed infection with asexual stages of *P. vivax*, written informed consent by the patient or attending parent/guardian, and a negative urine pregnancy test in women of childbearing age. Exclusion criteria were any clinical or laboratory feature of severe malaria [22], haemoglobin concentration of less than 7 g/dl, concomitant disease that would mask treatment responses, known allergy to any of the study drugs, anti-malarial treatment in the previous month, mixed species *Plasmodium *infection, pregnancy (or unwillingness to undergo pregnancy testing), lactation, and anticipated inability or unwillingness to complete the 56 day follow-up.

### Randomization

Patients were allocated to the two treatment arms based on a pre-generated randomization list made in blocks of 20 that was produced and held independently of the field teams by a statistician. The individual allocations were kept in sealed, opaque envelopes and opened only after enrolment by the field team. Patients and clinical field workers were, therefore, not blinded to the treatment arm after allocation. Microscopists were blinded to treatment allocation at follow-up examinations.

### Drugs

Patients received either quality-controlled chloroquine (IDA, Netherlands) aiming for a target total dose of 25 mg base/kg in divided doses over three days, or dihydroartemisinin-piperaquine (Artekin^®^, each tablet containing 40 mg dihydroartemisinin and 320 mg piperaquine; Holleypharm, China) aiming for a target total dose of 6/48 mg/kg in divided doses over three days. According to the body weight, individual doses were either rounded to the nearest half or quarter tablet or, if necessary, tablets were crushed, mixed with 5 ml water and the appropriate volume was given. All doses were taken under supervision at the malaria treatment centre and patients were observed for 60 minutes after drug administration. Patients vomiting within this time received the same dose again. Patients who vomited the medication twice were withdrawn from the study.

### Follow-up

The subjects were seen on days 0 to 3 inclusive and weekly to 56 days, so that late recrudescence and relapses could be detected [23,24]. Patients were also requested to attend if they felt unwell on any other day during the 56 days of follow-up. At each follow up visit a clinical assessment was undertaken and the patient was asked about adverse events by the use of a standard symptom questionnaire. Thick and thin blood slides were examined and haemoglobin was measured. Other tests such as peripheral white blood cell count and urine examination were undertaken as indicated to rule out other conditions and investigate possible adverse effects of drugs. Patients showing parasitological failure with recurrence of *P. vivax *had capillary blood collected on filter paper for PCR analysis and were treated with chloroquine (25 mg base/kg over 3 days) and followed up by standard protocols. Patients with mixed *P. falciparum/P. vivax *infection were also classified as treatment failures but treated with artesunate/sulphadoxine-pyrimethamine. Patients were censored from the analysis if there was stated withdrawal of consent at any stage, persistent vomiting during the acute phase (necessitating parenteral treatment) or occurrence during the follow-up of concomitant disease that would interfere with a clear classification of the treatment outcome (including mono-infection with *P. falciparum*). Patients failing to attend follow-up visits received a visit at home and were asked to reattend the centre for assessment. Transportation was provided for all follow-up visits for all subjects. All enrolled patients were given one long-lasting insecticide treated bed net.

### Outcomes

The primary outcome was the difference in the proportion of patients with parasitologically-confirmed recurrence of *P. vivax *infection at or before day 56 between the two arms. Secondary outcomes were parasite and fever clearance times, recurrence at earlier time points, gametocytaemia at day 28 and 56, haemoglobin recovery, and safety and tolerability of the treatments.

### Sample size

Given the relatively high predicted efficacy of chloroquine, a non-inferiority design was used [23] with the aim of testing whether dihydroartemisinin-piperaquine was non-inferior to (i.e. equivalent or better than) chloroquine in terms of the proportion of patients with parasitological failure at 56 days; the non-inferiority margin (Δ) was set at 5%. The sample size was 550 patients (275 per arm), calculated assuming a 95% cure rate with chloroquine, a one-sided alpha of 0.05 and 80% power.

### Statistical analysis

Data were double entered into Microsoft Access. All analyses were conducted using STATA version 9.0. The Student's t-test, Mann-Whitney U and chi-squared (or Fisher's exact) tests were used for comparison of baseline variables, as appropriate. Parasitological failure rates were assessed by survival analysis using the Kaplan-Meier method and Wilson confidence intervals for the difference in efficacy proportions were calculated using the effective sample size [25]. Dihydroartemisinin-piperaquine was considered non-inferior to chloroquine if the lower bound of the confidence interval of the difference was greater than 0.05 (the non-inferiority margin). Differences between treatment groups were estimated using the log-rank test. A sensitivity analysis, in which losses to follow-up were considered failures, was also undertaken [26]; patients who did not complete treatment were excluded. Cox regression including age (years), gender, recruitment site, treatment arm, admission haemoglobin level (g/dl), presence of gametocytes and parasitaemia as independent variables was performed to identify possible independent predictors of recurrence, using a stepwise elimination method.

### Species confirmation by PCR

DNA was extracted from filter-paper blood spots and nested PCR performed to detect *P. vivax *and *P. falciparum *as described previously [27].

### Ethical approval

The study was approved by the Ethics Committee of the Faculty of Tropical Medicine, Mahidol University, Thailand, the Oxford Tropical Research Ethics Committee, Oxford University, UK and the Institutional Review Board of the Afghan Public Health Institute, Ministry of Public Health, Afghanistan. The trial was registered with the clinical trials website http://www.clinicaltrials.gov/ct as NCT00682578. This description of the trial describes recruitment of patients with uncomplicated infection with either *P. falciparum *or *P. vivax*, with treatments depending on the species detected. Recruitment of *P. falciparum *cases proved much slower than for *P. vivax *and the ongoing *P. falciparum *study will be presented elsewhere.

## Results

### Recruitment

**Figure 1:**
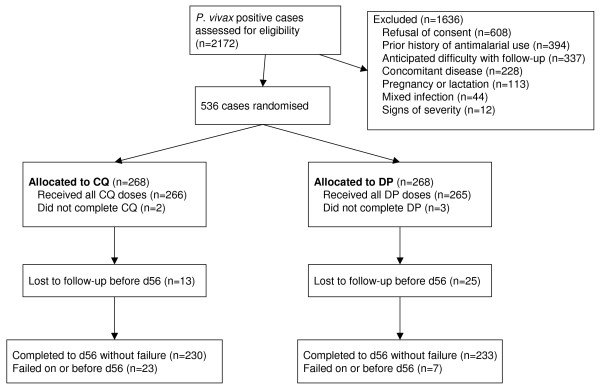
**Trial flow**.

From July 2007 to February 2009, 536 patients were enrolled in the study with 268 receiving chloroquine and 268 dihydroartemisinin-piperaquine (Figure 1). The main reasons for not being enrolled in the study were lack of consent (n = 608; 37%), history of taking anti-malarial drugs in the past month (n = 394; 24%), home too far away for follow-up (n = 337; 20%), concomitant disease (n = 228; 14%), pregnancy or lactation (n = 113; 7%), mixed infection with *P. falciparum *(n = 44) and severe malaria (n = 12). The majority of patients (314/536 (58.6%)) were recruited in Jalalabad; 119 (22.2%) patients were recruited in Taloqan and 103 (19.2%) in Maimana. 73 (13.6%) patients were less than five years of age, 258 (48.1%) were 5-14 years of age and 205 (38.2%) were 15 or older.

At the reference laboratory 94% of baseline slides were confirmed as *P. vivax *monoinfection; the remaining slides were classed as *Plasmodium *infection of undetermined species because of slide quality. PCR confirmation was undertaken on an independent, randomly selected subset of paired blood samples from 10 patients with parasitological failure. All 10 baseline samples and 9 of 10 samples taken at recurrence contained *P. vivax *by this method. *P. falciparum *was not detected in any sample by either microscopic or molecular methods.

### Baseline characteristics

Patient characteristics at baseline were broadly similar between the two treatment groups (Table 1) except that the median white blood cell count was lower in the chloroquine group compared with dihydroartemisinin-piperaquine (median 6.0 vs. 6.6 × 10^9^/l respectively, p = 0.02).

**Table 1:** Baseline characteristics of enrolled patients.

Characteristic	Treatment arm		
Number of patients randomized	CQ (n = 268)	DP (n = 268)	*P*
Gender: Male/Female (ratio)	126/142 (0.89)	132/136 (0.97)	0.60
Age, median years (range)	11 (0.25 -71)	12 (1 - 70)	0.38
			
Age Groups:			0.18
<5 years	43 (16.0)	30 (11.2)	
5 -14 years	130 (48.5)	128 (47.8)	
>14 years	95 (35.5)	110 (41.0)	
			
Location:			0.61
Jalalabad	154 (57.5)	160 (59.7)	
Taloqan	58 (21.6)	61 (22.8)	
Maimana	56 (20.9)	47 (17.5)	
			
Weight, median kg (range)	30 (5-93)	33 (7-89)	0.69
Geometric mean parasitaemia/μL (95% CI)	3339 (3000-3716)	3236 (2940 -3560)	0.67
Number with gametocytes	218 (81.3)	203 (75.2)	0.12
Haemoglobin concentration, mean g/dl (SD)	10.9 (1.46)	10.9 (1.42)	0.99
Anaemia (haemoglobin concentration < 10 g/dl)	49 (18.4)	44 (16.5)	0.35
Median white blood cells ×10^9^/ml, median (range)	6.0 (3.6-54)	6.6 (3.0-12.4)	0.02
Body temperature °C, mean (95% CI)	37.3 (37.2-37.4)	37.3 (37.2-37.4)	0.77
Median systolic blood pressure, mmHg (range)	100 (80-160)	100 (80-170)	0.43
Median diastolic blood pressure, mmHg (range)	70 (50-100)	70 (50-100)	0.38
Median pulse rate, beat/min (range)	88 (60-140)	88 (60-140)	0.53
Jaundice	30 (11.3)	32 (12.1)	0.79
History of convulsions in last 24 hours	1 (0.37)	1 (0.37)	

Data for chloroquine (CQ) and dihydroartemisinin-piperaquine (DP) are number (%) unless otherwise indicated.

### Response to treatment and follow-up

Patients in the chloroquine group who completed treatment received a median total dose of 25.9 mg/kg base (IQR 25.0 - 27.3 mg/kg); for the dihydroartemisinin-piperaquine group the median total dihydroartemisinin dose was 6.0 mg/kg (IQR 5.45 - 6.67 mg/kg). Both treatments were generally well tolerated. Repeated vomiting of the study medication was not reported within 60 minutes of drug administration in any patient. No patient deteriorated or developed signs of severity. Five patients who received the first dose withdrew consent before completing treatment and were censored from analysis. Of the 531 individuals who completed treatment, 38 (7.2%) cases were lost to follow-up before day 56; 13 in the chloroquine arm and 25 in dihydroartemisinin-piperaquine arm (p = 0.04) (Figure 1).

### Parasitological failures

**Figure 2:**
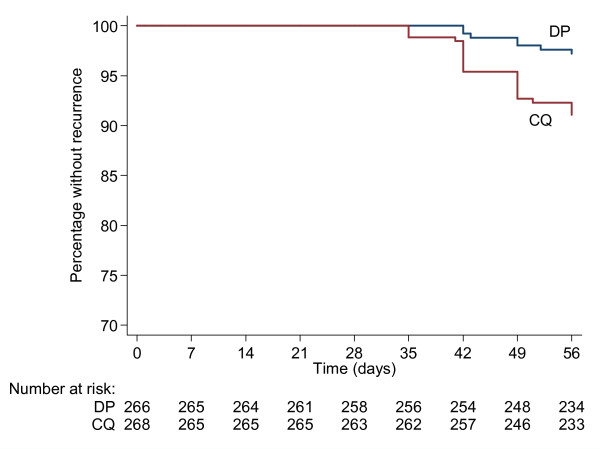
**Survival curves**. The proportion of subjects free from recurrence of *P. vivax *is displayed according to treatment arm; dihydroartemisinin-piperaquine (DP) and chloroquine (CQ).

**Figure 3:**
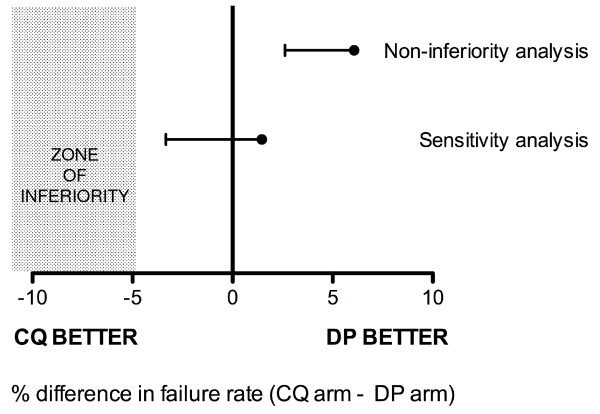
**Analysis of primary outcome (cumulative failure rate at day 56)**. Non-inferiority analysis refers to the pre-specified survival analysis.

By the end of the 56-day follow-up period, there were 23 recurrences (parasitological failures) in the chloroquine group and 7 in the dihydroartemisinin-piperaquine group, giving a day 56 failure rate of 8.9% (95% CI 6.0 - 13.1%) in the chloroquine group and 2.8% (1.4 - 5.8%) in the dihydroartemisinin-piperaquine group (Figure 2). The difference in day 56 parasitological failure rates between chloroquine and dihydroartemisinin-piperaquine was 6.1% (2-sided 90% CI +2.6 to +9.7%). The lower bound of this confidence interval was not only higher than the prespecified non-inferiority margin (i.e. -5%), but also did not include zero, indicating that dihydroartemisinin-piperaquine was superior to chloroquine in terms of outcome (Figure 3) [26]. The superiority of dihydroartemisinin-piperaquine was confirmed by the log rank test (p = 0.003).

A sensitivity analysis (in which all losses to follow-up were classed as failures) yielded a difference in cumulative parasitological failure rate between chloroquine and dihydroartemisinin-piperaquine of 1.5% (2-sided 90% CI -3.3 to +6.3%); as predicted there was a wider 90% confidence interval than that based on survival analysis but also a lower point estimate for the difference in failure rate between the chloroquine and dihydroartemisinin-piperaquine groups caused by the greater number of losses to follow-up in the dihydroartemisinin-piperaquine group. The lower bound of the 90% confidence interval was still higher than the prespecified non-inferiority margin (5%), but included zero, indicating that dihydroartemisinin-piperaquine was not inferior to chloroquine even in this considerably more conservative analysis [26] (Figure 3).

### Secondary outcomes

**Figure 4:**
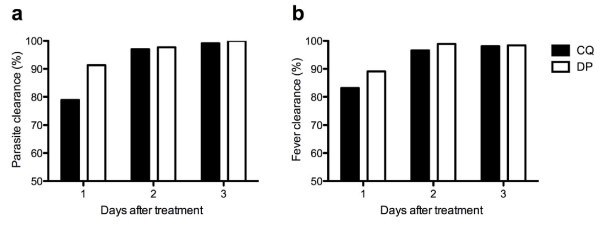
**Proportion with clearance of parasites (a) and fever (b) at day 1-3 after treatment**.

Initial parasitological responses were faster with dihydroartemisinin-piperaquine (Figure 4); in the dihydroartemisinin-piperaquine group 241/264 (91.3%) of patients cleared their parasitaemia at day 1 compared to 209/265 (78.9%) in the chloroquine group (p < 0.001, relative risk of clearance in chloroquine group = 0.87, 95% CI 0.81-0.93). At day 2, the groups were no longer significantly different with 259/265 (97.8%) of the dihydroartemisinin-piperaquine group and 257/265 (97.0%) in the chloroquine group having cleared their parasitaemia (p = 0.59). Two patients in the chloroquine arm remained parasitaemic at day 3.

In terms of resolution of fever (defined as axillary temperature >37.0°C) dihydroartemisinin-piperaquine was again superior (Figure 4), with 29/266 (10.9%) of the dihydroartemisinin-piperaquine group febrile on day 1 compared to 45/268 (16.8%) in the chloroquine group (p = 0.049). By day 2 there was no longer a significant difference in the proportion with fever (3/265 (1.1%) for dihydroartemisinin-piperaquine, 9/264 (3.4%) for chloroquine, p = 0.08). At day 1, headache was less frequent in the dihydroartemisinin-piperaquine arm (105/265 (39.6%)) compared to the chloroquine arm (145/266 (54.5%), p = 0.001).

The day 28 cure rate was 100% in both arms with the first recurrences observed at day 35 in the chloroquine group and day 42 in the dihydroartemisinin-piperaquine group. Twenty-one of 30 (70%) recurrent parasitaemias were associated with gametocytaemia, with no significant difference between treatment arms in terms of proportion of recurrent patients with gametocytaemia (16/23 and 5/7 for chloroquine and dihydroartemisinin-piperaquine respectively, p = 1.0).

### Adverse events

There were no serious adverse events recorded during the trial and no patient required hospitalization. Both study drugs appeared to be well tolerated. There was a significant increase in the proportion of patients with pruritus in the chloroquine arm compared to dihydroartemisinin-piperaquine on days 1, 2 and 3; on day 1, 15/268 subjects in the chloroquine arm had pruritus compared to 2/266 in the dihydroartemisinin-piperaquine arm (p = 0.002, relative risk 7.44, 95% CI 1.71-32.3), consistent with the known side-effects of chloroquine. None of the other signs or symptoms documented in the first three days of treatment were found to be significantly different between the groups. There were no significant differences between the two arms in haemoglobin concentration or occurrence of anaemia (haemoglobin < 10 g/dl) at any stage during follow-up (all p > 0.5).

### Predictors of treatment failure

A univariate analysis undertaken to identify variables associated with failure showed that treatment allocation (chloroquine rather than dihydroartemisinin-piperaquine), study site (recruitment in Maimana compared to Jalalabad), younger age and lower haemoglobin concentration at baseline were all significantly associated with recurrent infection. Admission parasitaemia, presence of gametocytes at admission and gender had no effect in this model (Table 2).

**Table 2:** Univariate analysis of risk factors for treatment failure

	Univariate analysis	
Factor	Hazard Ratio	p value
Chloroquine treatment	3.28 (1.41-7.63)	0.006
Female	0.93 (0.46-1.91)	0.85
Age (years)	0.96 (0.92-1.00)	0.03
Recruitment site (Jalalabad as reference)		
Taloqan	0.79 (0.26-2.43)	0.68
Maimana	3.06 (1.42-6.61)	0.004
Parasitaemia	0.86 (0.32-2.29)	0.76
Presence of gametocytes	1.13 (0.46-2.77)	0.784
Haemoglobin at admission (g/dl)	0.49 (0.37-0.65)	<0.001

Multivariate analysis of the same seven variables entered into the univariate analysis indicated that treatment with chloroquine and lower haemoglobin concentration at baseline were the only two significant independent predictors of recurrent infection (Adjusted Hazard Ratio (AHR) 3.66, 95% CI 1.48-9.02, p < 0.001 for treatment allocation and AHR 0.50, 95% CI 0.37-0.66, p = 0.005 for haemoglobin concentration).

## Discussion

This trial in three separate malaria areas of Afghanistan confirmed that both chloroquine and dihydroartemisinin-piperaquine are well-tolerated and broadly efficacious treatments for vivax malaria. Initial parasite clearance was significantly faster after dihydroartemisinin-piperaquine, consistent with the pharmacodynamic properties of artemisinin-containing therapies observed in *P. falciparum *[28] and *P. vivax *[9,24]. Nevertheless 97% of patients in the chloroquine arm were parasite free and afebrile by day 2, and there were no recurrences in either arm before day 28, suggesting that *P. vivax *remains sensitive to chloroquine in the study area, which included locations both north and south of the Hindu Kush divide of central Afghanistan. These data extend the geographic range of efficacy monitoring beyond Eastern Afghanistan where chloroquine has been shown previously to be efficacious in clearing *P. vivax *parasitaemia [9,10]. These findings stand in marked contrast to data from Indonesia where *P. vivax *chloroquine-resistance manifests as frequent failure to clear parasites by day 4 [6,29-31] and recurrence rates by day 28 approximate 50% [29-31] or higher [6].

Over the 56-day follow-up period dihydroartemisinin-piperaquine proved superior to chloroquine in terms of the prespecified survival analysis of recurrence rates. Approximately 9% of cases had recurrent *P. vivax *infections in the chloroquine arm, compared to 3% in the dihydroartemisinin-piperaquine arm, with all failures occurring on or after day 35. Recurrences of *P. vivax *may consist of a mixture of relapses from liver hypnozoites, recrudescences of the erythrocytic infection (due to inadequate drug levels or resistance), and reinfections acquired from additional innoculations. It is not possible with current methodologies to distinguish reliably between these possibilities [32,33]. However, in studies undertaken in a neighbouring region of Pakistan, radical treatment with primaquine following chloroquine prevented the majority of recurrent infections over the course of 9-12 months, indicating that most recurrences following chloroquine monotherapy are likely to be relapses from liver hypnozoites [34-36]. In this study, primaquine was not given, and none of the treatments administered has activity against *P. vivax *hypnozoites. The rapid clearance of parasites in the chloroquine group, and the fact that failures were not seen before day 28, suggest that recrudescences associated with chloroquine-resistance did not contribute significantly to the number of recurrences. For these reasons, the majority of recurrences observed in this study are likely to have been relapses.

The pattern of recurrences is consistent with the known properties of the drugs under study. Chloroquine is effective in preventing early relapses (<28 days) of vivax malaria in chloroquine-sensitive vivax malaria [4,9,10,36-39]. This is because chloroquine is eliminated very slowly (terminal half-life one to two months), and provides post-treatment prophylaxis against relapse for 3-4 weeks after treatment [39]. Piperaquine also has a very long terminal elimination half- [40]. In an area where chloroquine-resistant *P. vivax *is prevalent DHA-PPQ has been shown in several comparative trials to prevent recurrences of *P. vivax *over a six-week period more effectively than ACT containing partner drugs with shorter half-lives [5,14,15]. In these studies, the recurrence rate (typically measured over 42 days) can be correlated with the half-life of the partner drug; for example, recurrences are fewer in patients treated with dihydroartemisinin-piperaquine compared to artemether-lumefantrine [5,14] and artesunate-amodiaquine [15], presumably because the lumefantrine and amodiaquine components have shorter terminal elimination half-lives (approximately 10 and 18 days respectively) than piperaquine.

In the only previous trial directly comparing dihydroartemisinin-piperaquine to chloroquine (combined with sulfadoxine-pyrimethamine, CQ-SP) for *P. vivax *infection in Papua New Guinea, high levels of chloroquine-resistance were present, as evidenced by prolonged parasite clearance times in the CQ-SP arm. There was an 87% rate of recurrence in the first 42 days after CQ-SP compared to 30.6% in the dihydroartemisinin-piperaquine arm [5], a difference that is likely to reflect both failure to clear initial parasitaemia (leading to recrudescence) as well as failure to suppress relapse. This study provides the first comparison of chloroquine and dihydroartemisinin-piperaquine in chloroquine-sensitive *P. vivax*, and confirms the longer lasting post-treatment prophylactic effect of dihydroartemisinin-piperaquine compared to chloroquine in this setting. The observed proportion of chloroquine-treated patients with recurrence over 56 days is less than in a study undertaken in the Jalalabad Malaria Reference Centre in 2004 [9] (where more than 40% of patients treated with chloroquine had recurrence from days 28-42 after treatment), for reasons that are unclear. Ancillary studies would help to shed further light on these issues, including pharmacological assessment of chloroquine and piperaquine levels in blood during the follow-up period. This would also allow the question of chloroquine resistance to be excluded more definitively.

This study was not powered to undertake detailed assessments of factors associated with recurrence. In the univariate analysis (although not the multivariate), younger age was found to be a risk factor for recurrence, a finding consistent with previous studies examining this effect over longer follow-up periods [35,37]. Possible explanations for this include pharmacokinetic effects [39,41], reduced immunity compared to adults and higher burdens of hypnozoites. Patients recruited in Maimana had a significantly higher rate of failure than those from Jalalabad in the univariate analysis although the effect disappeared in the multivariate analysis, suggesting that other factors may have contributed to this finding. The data do not provide substantive support for the existence of chloroquine resistance at Maimana or any of the study sites. The finding of a strong association between lower baseline haemoglobin and recurrence of *P. vivax *infection was not anticipated and requires further investigation.

It is possible that the action of dihydroartemisinin-piperaquine is simply to delay relapses of vivax malaria rather than prevent them; in other words the survival curves for recurrence with dihydroartemisinin-piperaquine and chloroquine will eventually merge. Studies with longer-term follow-up would be needed to determine if this is the case and if dihydroartemisinin-piperaquine offers tangible benefits in terms of long-term health of the individual (preventing relapse and associated anaemia) and possibly the community (by reducing transmission). Any health benefits have to be balanced against the relative cost of each treatment course, which is currently in favour of chloroquine use. These issues also have to be considered in the context of the challenges of treating patients with radical therapy in this region [35,36].

## Conclusion

Both dihydroartemisinin-piperaquine and chloroquine are efficacious treatments for *P. vivax *malaria across three regions of Afghanistan. Consistent with the known pharmacological properties of the drugs, dihydroartemisinin-piperaquine provides a longer period of post-treatment prophylaxis, reducing the incidence of recurrence from 4-8 weeks after treatment.

## Competing interests

The authors declare that they have no competing interests.

## Authors' contributions

GRA, SP, MI, AMD, SJL, NPJD, PS, NJW and FK were involved in the conception and design of the study. GRA and FK were responsible for supervising patient recruitment and follow-up. MI undertook species confirmation by PCR. GRA, SP, CJW and SJL participated in the statistical analysis. GRA, SP, CJW, SJL and NJW drafted and critically revised the manuscript. All authors read and approved the final manuscript.
